# Compensating the effect of dendritic diameters on calcium transients: a modeling study

**DOI:** 10.1186/1471-2202-12-S1-P60

**Published:** 2011-07-18

**Authors:** Haroon Anwar, Erik De Schutter

**Affiliations:** 1 Computational Neuroscience Unit, Okinawa Institute of Science and Technology, Okinawa 904-0411, Japan; 2 Theoretical Neurobiology, University of Antwerp, B-2610 Antwerpen, Belgium

## 

Intracellular Ca^2+^ does not only play a crucial role in the physiological interaction between the Ca^2+^ channels and Ca^2+^ activated K^+^ channels, it also serves as an important cellular messenger in signaling pathways. Therefore, accurate representation of intracellular calcium concentration is required in biophysically plausible models. Most commonly, intracellular calcium is modeled in morphologically realistic neuron models using single [1-3] or double exponential decaying pools [4] where Ca^2+^ concentration is computed in a submembrane shell only. These models are rather insensitive to the diameter of a compartment but fail to simulate interaction between Ca^2+^ channels and Ca^2+^ activated K channels occurring at multiple time scales. A more comprehensive and biophysically realistic solution is to use a detailed calcium dynamics model [5,6] with buffers, pump and diffusion. When we used detailed Ca^2+^ dynamics model with a detailed morphology of a Purkinje cell, we discovered large gradients of Ca^2+^ levels in neighboring segments with different diameters even in the present of lateral diffusion. The peak Ca^2+^ concentration showed a close to linear inverse relationship to diameter of the compartment. We deem such pronounced gradients of Ca^2+^ as unphysiological and suggest that there should be a regulatory mechanism to compensate the effect of local dendritic geometry on buffered calcium transients.

In this study, we used a detailed calcium dynamics model [6] in a piece of dendrite where diameters of segments were varied to study different combinations of diameter changes (small to large variation). All the simulations were run using STEPS [7] with a fine resolution mesh to allow accurate modeling of diffusion.

Assuming a uniform calcium channel density for influx and uniformly distributed calcium buffers and pumps, difference in diameters of segments gave rise to large gradients of calcium transients. We investigated several possible mechanisms that could compensate the effect of local dendritic geometry on gradients of intracellular levels. These regulatory mechanisms included scaling of channel densities, scaling of pump densities, scaling of buffer concentrations and subcellular localization of buffers. Further, we also investigated combination of these regulatory mechanisms to compensate for the differences in peak amplitudes of calcium transients.

Our results suggest that the effect of local dendritic geometry on intracellular calcium levels can be partially compensated by each of the regulatory mechanisms investigated and can be sufficiently compensated by combination of those regulatory mechanisms. Therefore, on the basis of our modeling work, we propose a quantitative physiological investigation of the suggested mechanisms.
